# Effect of *Rhizopus nigricans* (*Rhizopus stolonifera*)-based novel starter culture on quality and safety attributes of *doenjang*, a traditional Korean soybean fermented food product

**DOI:** 10.1038/s41598-019-57382-y

**Published:** 2020-01-23

**Authors:** Shruti Shukla, Ashutosh Bahuguna, Hae-Kyong Park, Jong-Kyu Kim, Myunghee Kim

**Affiliations:** 1 Department of Food Science and Technology, National Institute of Food Technology Entrepreneurship and Management (NIFTEM), Sonipat, 131028 Haryana India; 2 Department of Food Science and Technology, Yeungnam University, Gyeongsan-si, Gyeongsangbuk-do, 38541 Republic of Korea

**Keywords:** Fungi, Fungal pathogenesis, Food microbiology

## Abstract

We aimed to develop a consortium of starter culture of effective microorganisms to prepare *doenjang*, a traditional Korean fermented food. Different ratios of *Bacillus subtilis* TKSP 24 (B), *Aspergillus oryzae* complex (A), *Rhizopus nigricans* (also named as *Rhizopus stolonifera*) (R), and *Mucor racemosus* 15 (M15) were selected as *meju* starter cultures to produce *doenjang* with improved quality. Microbial strain combinations (B: A: R and B: M15: R) were mixed separately at three different ratios [1:1:1 (w/w), 1:0.5:1.5 (w/w), and 1:1.5:0.5 (w/w)] to prepare BAR-1, BAR-2, BAR-3, BM15R-1, BM15R-2, and BM15R-3 *doenjang* samples. Quantitative analyses included free amino acids, free sugar, volatile and non-volatile organic acids, cellular antioxidant activity along with the presence of biogenic amines and aflatoxins, and microbial counts. Total free amino acids responsible for the sweet taste of *doenjang* were highest in BAR-2 (322.50 mg/100 g) and BM15R-3 (320.07 mg/100 g). Total volatile organic acid was highest in BAR-1 compared to other preparations. All *doenjang* samples had biogenic amines, especially histamine, below the toxicity level (500 mg/kg). Also, the aflatoxin and hazardous microbial count in the tested *doenjang* samples were below the level of toxicity. The findings suggest that use of multiple microbial strains in combination with *R. nigricans* as a starter culture could be a novel and effective approach to improve the nutrition and safety of fermented soybean food products of *doenjang*.

## Introduction

*Doenjang* is a protein-rich traditional food product and flavouring ingredient in Korea that is produced by the fermentation of soybeans. Traditionally, in the preparation of starter culture (*meju*) for *doenjang*, soybeans are fermented by *Bacillus* species in the early stage, followed by the secondary microflora of *Aspergillus* species such as *Aspergillus oryzae* and *Aspergillus niger*^1^. Processing of traditional *doenjang* differs depending on its region of origin^1^. In addition, traditional *meju* can be made from various salt solutions and is fermented for more than 2 months^2^. The taste and colour of *doenjang* are considered as the main factors responsible for its overall acceptability, which is highly influenced by the starter culture and the presence of oxygen^1^. A standardised starter culture would be advantageous to ensure the rapid production of *doenjang* with improved quality and safety.

Several microorganisms, mainly filamentous fungi including *Rhizopus* species, *Bacillus* species, and lactic acid bacteria are commonly found during the fermentation of soybeans^3^. *Rhizopus* species have an especially vital role in the food industry due to their ability to produce thermostable polygalacturonases, which hydrolyses α-1,4 glycosidic bonds between galacturonic acid residues and thus affect the plant cell wall^4^. In particular, *R. oligosporus* is used to ferment soybeans to make *tempeh*, which has many health benefits including potent anti-radical activity^5^. *R. nigricans* (also named as *Rhizopus stolonifera*) ferments the ground nut flour, which improves its nutritional value^6^. *R. nigricans* has received increased interest in the fermentation industry because of its ability to produce fumaric acid abundantly, which has acidifying and gelling properties^7,8^.

Safety concerns with *doenjang* include microbial contamination, formation of biogenic amines, and production of aflatoxins, which depend on various factors, such as raw materials, microbial flora, fermentation conditions, and salt content^9^. Several studies have been carried out to produce commercially modified traditional Korean *doenjang*^1^. However, no studies have so far been carried out on commercially modified *meju* prepared by the inoculation of a mixture of various starter cultures, especially *Rhizopus* strains. The present study sought to develop a novel starter culture in combination with *R. nigricans* for the preparation of *doenjang*. Prepared *doenjang* samples were evaluated for their nutritional and safety parameters. Additionally, the cellular antioxidant activity of the selected *doenjang* sample was carried out to demonstrate its potential against oxidative stress induced by various reactive oxygen species (ROS).

## Materials and Methods

### Chemicals and reagents

All chemicals and solvents were of the analytical and chromatographic grade. Standard biogenic amines (agmatine sulfate, tryptamine hydrochloride, 2-phenylethylamine, putrescine dihydrochloride, cadaverine dihydrochloride, histamine dihydrochloride, tyramine hydrochloride, spermidine trihydrochloride, and spermine tetrahydrochloride), dansyl chloride, and acetone were purchased from Sigma-Aldrich (St. Louis, MO, USA). Sodium hydroxide, sodium hydrogen carbonate, ammonium hydroxide, and perchloric acid were purchased from Junsei Chemicals (Tokyo, Japan). High-performance liquid chromatography (HPLC) grade acetonitrile and ammonium acetate were obtained from Merck (Darmstadt, Germany). Nutrient agar was purchased from Difco (Sparks, MD, USA). API kits were purchased from BioMerieux (Marcy I’ Etoile, France).

### Microbial strains

*Bacillus subtilis* TKSP 24, *A. oryzae* complex, *Mucor racemosus* 15, and *R. nigricans* were isolated from traditional *meju* and identified using 16 S rRNA and internal transcribed spacer gene sequence analyses. All microorganisms were grown individually under aseptic conditions and used as a starter culture. The microbial strains were deposited in National Center for Biotechnology Information (NCBI) GenBank and Korean Collection for Type Cultures (KCTC), Republic of Korea under the following accession numbers: *B. subtilis* TKSP 24 (KCTC 11840BP; NCBI GenBank accession No. MN784431), *A. oryzae* complex (KCTC 11841BP; NCBI GenBank accession No. MN784429), *M. racemosus* 15 (KCTC 11842BP; NCBI GenBank accession No. MN784430), *R. nigricans* (*R. stolonifera*) (KCTC 12107BP; NCBI GenBank accession No. MN791090).

### Preparation of *meju* and *doenjang*

Previously prepared starter cultures were mixed separately with boiled soybeans at a concentration of 1% (w/w) on a stainless-steel square plate (36 cm × 32 cm × 6 cm). At 70% relative humidity, *A. oryzae* complex, *M. racemosus* 15, and *R. nigricans* seeded soybeans were incubated for 3 days at 28 °C. They were designated A *meju*, M15 *meju*, and R *meju*, respectively. Soybeans seeded with *B. subtilis* TKSP 24 were incubated at 30 °C for 2 days and named as B *meju*. Six different *doenjang* samples were prepared by mixing these four starter cultures in different proportions. *Doenjang* samples, BAR-1, BAR-2, and BAR-3, were prepared by mixing *B. subtilis meju*, *A. oryzae* complex *meju*, and *R. nigricans meju* at 1:1:1 (w/w), 1:0.5:1.5 (w/w), and 1:1.5:0.5 (w/w), respectively. Similarly, *doenjang* samples, BM15R-1, BM15R-2, and BM15R-3, were prepared by mixing *B. subtilis meju*, *M. racemosus* 15 *meju*, and *R. nigricans meju* at 1:1:1 (w/w), 1:0.5:1.5 (w/w), and 1:1.5:0.5 (w/w), respectively (Table 1). All *meju* preparations were fermented at 30 °C for 30 days in the presence of a 15% salt solution^10^. After 30 days of fermentation, the solid portion was separated from the liquid and re-fermented at 30 °C for 30 days to obtain the final *doenjang* product. Traditional Korean *doenjang* prepared with natural microflora served as a control. The above described *doenjang* and *meju* production processes were repeated several times to confirm reproducibility.

**Table 1:** List of *doenjan*g samples fermented with various *meju* products and their pH values.

Four strains used for *meju* preparation	Mixing ratios of each *meju* preparation (w/w)	Code of *doenjang* samples made from various combinations	pH^b^ of *doenjang* samples
*B. subtilis* TKSP 24 *A. oryzae* complex *R. nigricans M. racemosus* 15	*B. subtilis* TKSP 24 (1) : *A. oryzae* complex (1) : *R. nigricans* (1)	BAR-1	4.97 ± 0.01
	*B. subtilis* TKSP 24 (1) : *A. oryzae* complex (0.5) : *R. nigricans* (1.5)	BAR-2	5.20 ± 0.03
	*B. subtilis* TKSP 24 (1) : *A. oryzae* complex (1.5) : *R. nigricans* (0.5)	BAR-3	5.32 ± 0.01
	*B. subtilis* TKSP 24 (1) : *M. racemosus* 15 (1) : *R. nigricans* (1)	BM15R-1	5.49 ± 0.00
	*B. subtilis* TKSP 24 (1) : *M. racemosus* 15 (0.5) : *R. nigricans* (1.5)	BM15R-2	5.27 ± 0.11
	*B. subtilis* TKSP 24 (1) : *M. racemosus* 15 (1.5) : *R. nigricans* (0.5)	BM15R-3	5.33 ± 0.01
Traditional *meju*	Natural microflora	Control^a^	5.60 ± 0.11

^a^Traditional Korean *doenjang* made of natural microflora. ^b^pH expressed in mean values of triplicate.

#### Measurement of pH

The pH levels of *doenjang* samples was determined as previously described method^2^ using a pH meter (Thermo Electroncorporation; Beverly, MA, USA).

#### Colour analysis

The colour values of *doenjang* samples was determined by using a chromameter (Minolta; Osaka, Japan). *Doenjang* samples were placed on a white surface and the Hunter L*, a*, and b* colour values were measured as previously described method^11^.

### Analysis of taste components

#### Sample preparation

A previously described method^12^ was adopted for the extraction of free amino acids, free sugars, and organic acids (volatile and non-volatile) from *doenjang* samples. Briefly, 100 g of *doenjang* was refluxed with 150 ml of 85% ethanol at 65 °C for 1 h, followed by filtration. The residual was re-extracted twice using same conditions. The collected filtrates were pooled and centrifuged at 4000 × *g* for 20 min at 4 °C. The supernatant was collected and vacuum-dried.

#### Quantification of free sugar contents

Free sugars were separated by passing the extracted *doenjang* samples on ion exchange column as previously described^12^. Amberlite IR-120 and amberlite IRA-400 columns were used as cation and anion exchangers for chromatographic separation, respectively. Quantitative analysis of the eluted free sugars was carried out by HPLC using a model 600E HPLC unit (Waters; Milliford, MA, USA), equipped with an RI-Model 410 refractive index detector. Separation was achieved using a Sugar-Pak I column (6.5 mm internal diameter × 300 mm length; Waters) using Ca-EDTA buffer solution (50 mg of Ca-EDTA per litre of distilled water) as the mobile phase at a constant flow rate of 0.5 ml/min. Free sugars were identified and quantified in *doenjang* samples by comparison with standard sugar samples of known strength.

### Quantification of free amino acids and non-volatile organic acids

Quantification of free amino acids and non-volatile organic acids in *doenjang* samples were carried out by the method adopted by Shukla *et al*.^12^. Briefly, the amberlite IR-120 and IRA-400 columns used earlier for free sugar collection were washed with 2 N NH_4_OH and 2 N (NH_4_)_2_CO_3_ for the recovery of bound free amino acids and non-volatile organic acids. Non-volatile organic acids were quantified by HPLC using an RS Pak KC-811column (8.0 mm internal diameter × 300 mm length) at a constant column temperature of 40 °C. H_3_PO_4_ solution (0.1% in distilled water) was used as a mobile phase with a constant flow rate of 1 ml/min.

Separation and quantification of amino acids was made using an amino acid analyser (Hitachi; Tokyo, Japan), equipped with a cation exchange resin in a column (4.6 mm internal diameter × 60 mm length) at a temperature of 30 °C to 70 °C as a method adopted by Shukla *et al*.^12^.

#### Quantification of volatile organic acids

Extraction and quantification of volatile organic acids were carried as previously described method^13^. In brief, 2 µl of each extracted *doenjang* sample and standard were separately injected into a gas chromatography system (Hewlett-Packard; Palo Alto, CA, USA). Separation was carried out in a glass column filled with 10% polyethylene glycol 6000 (Shimazu; Tokyo, Japan) using helium as a carrier gas at a constant flow rate of 0.9 ml/min. The column was maintained at 170 °C throughout the operation process.

### Determination of biogenic amines

#### Extraction of biogenic amines

For extraction of biogenic amines, 5 g of *doenjang* sample was homogenized with 10 ml of 0.4 M perchloric acid, followed by centrifugation at 4000 × *g* for 10 min. The supernatant was collected, and the pellet was re-extracted twice with 10 ml of 0.4 M perchloric acid. All the collected supernatant was pooled and processed for derivatisation before HPLC analysis.

#### Derivatisation of biogenic amines

Derivatisation of biogenic amines was carried out by mixing 1 ml of extracted *doenjang* sample with 0.2 ml of 2 M NaOH, 0.3 ml of saturated NaHCO_3_, and 2 ml of 10 mg/ml dansyl chloride, followed by 45 min incubation at 40 °C. After incubation, the reaction was stop by adding 100 µl of 25% NH_4_OH, and the volume was adjusted to 5 ml by acetonitrile, followed by centrifugation at 2,500 × *g* for 5 min. Finally, samples were filtered through a 0.2 µm syringe filter (Woongki Science Co.; Seoul, Korea) and processed for HPLC analysis.

#### Separation and quantification of biogenic amines

Separation of biogenic amines was carried out using HPLC (Agilent Technologies; Santa Clara, CA, USA), equipped with an ultraviolet-visible spectrophotometer as a detector. A 20 µl volume of derivatised extracted biogenic amines sample was injected into an Agilent C-18 column (4.6 mm internal diameter × 150 mm length, pore size of 5 µm). Separation was carried by gradient elution using 0.1 M ammonium acetate and acetonitrile mobile phase at a constant flow rate of 1 ml/min. Biogenic amines were analysed at 254 nm. Quantification and identification of biogenic amines in samples were made by comparison with reference standards.

### Cellular antioxidant potential

To evaluate the functional property of *doenjang* samples, cellular antioxidant activity was carried out using BAR-1 as a representative candidate. For the evaluation of cellular antioxidant activity, 10 g of BAR-1 *doenjang* was extracted with 100 ml distilled water, followed by filtration and freeze-drying to obtain the dry powder.

### Cell and cell culture

NIH 3T3 mouse fibroblast cells were purchased from American Type Culture Collection (Manassas, VA, USA). Cells were grown and maintained in Dulbecco’s modified Eagle’s medium [10% (v/v) fetal calf serum and 1% (v/v) penicillin-streptomycin mix] in a 5% CO_2_ incubator at 37 °C.

### Cell viability assay

Effect of BAR-1 *doenjang* on the cell viability of NIH 3T3 cells was evaluated by the 3-(4,5-dimethylthiazol-2-yl)-2,5-diphenyltetrazolium bromide (MTT) assay^14^ using different concentrations of BAR-1 (0–10 mg/ml).

### Determination of cellular ROS

Cellular ROS were determined by 2′,7′-dichlorofluorescein diacetate (DCFDA) assay^15^. In brief, NIH 3T3 cells were grown in 24-well culture plates, followed by the treatment with various concentrations of BAR-1 (0 mg/ml, 0.5 mg/ml, 1.0 mg/ml, and 2.5 mg/ml). After 24 h incubation, oxidative stress in the cells was induced by treatment with 0.25 mM hydrogen peroxide (H_2_O_2_) for 2 h. Finally, cells were washed twice with 0.1 M phosphate buffered saline (PBS, pH 7.4), followed by the addition of DCFDA (20 µM). After 30 min of incubation, the stained cells were washed with 0.1 M PBS and immediately visualised by fluorescence microscopy (Nikon; Tokyo, Japan). The fluorescence intensity was quantified using Image J software (NIH; Bethesda, MD, USA).

### Analysis of microbial counts

Based on profiling of amino acids, volatile and non-volatile organic acids, and sugar derivatives, two *doenjang* samples (BAR-1 and BM15R-1) were selected as representatives and analysed for microbial food pathogens. Pathogenic microbes were analysed as previously described methods^16^. The analysis was carried out for the presence or absence of foodborne pathogens, including total coliforms, *Staphylococcus aureus*, *Escherichia coli* O157, and *Salmonella* species, using selective culture-dependent methods. Isolates showing typical pathogen morphologies were further identified using a microbial pathogen detection API kit.

### Determination of total aflatoxin levels

Analysis of aflatoxins in *doenjang* samples of BAR-1 and BM15R-1 was performed according to the instructions of the manufacturer of the quantitative aflatoxin detection kit (Neogen Corp.; Lansing, MI, USA). Aflatoxin was extracted by homogenizing 10 g of each sample in 50 ml of 70% methanol, followed by filtration. Filtered samples were transferred into wells of an ELISA plate pre-coated with an antibody against aflatoxin. After sufficient antigen (aflatoxin)-antibody interaction, a chromogenic substrate was added, and the intensity of the colour was measured at 650 nm using ELISA reader (Tecan; Mannedorf, Switzerland). Quantification of aflatoxin was made using Veratox data reduction software (Neogen Corp.) by converting absorbance into concentration against reference standards.

### Statistical analyses

Results were presented as mean ± standard deviation (SD) of three independent experiments. Statistical analyses involved one-way ANOVA, followed by Duncan test for post-hoc analysis using IBM SPSS 19 program (SPSS Inc; Chicago, IL, USA). *P* < 0.05 was considered to be significantly different.

## Results and Discussion

### *Doenjang* production

Six different kinds of *doenjang* samples were prepared using *meju* starter culture, formed by four different types microorganisms (Table 1). The *doenjang* sample designated BAR was prepared by mixing different ratios of *meju* starter culture made up of *B. subtilis*, *A. oryzae* complex, and *R. nigricans*. While *doenjang* samples designated as BM15R were prepared by mixing *meju* starter culture comprised of *B. subtilis*, *M. racemosus* 15, and *R. nigricans*. Three different types of *doenjang* designated as BAR-1, BAR-2, and BAR-3 were prepared by mixing *B. subtilis meju*, *A. oryzae* complex *meju,* and *R. nigricans meju* at ratio of 1:1:1 (w/w), 1:0.5:1.5 (w/w), and 1:1.5:0.5 (w/w), respectively. Similarly, *doenjang* samples, BM15R-1, BM15R-2, and BM15R-3, were prepared by blending *B. subtilis meju*, *M. racemosus* 15 *meju,* and *R. nigricans meju* at a ratio of 1:1:1 (w/w), 1:0.5:1.5 (w/w), and 1:1.5:0.5 (w/w), respectively.

### Measurement of pH

pH is one of the most important parameters influencing proper fermentation and many biochemical events in fermented foods. The pH values of all the prepared *doenjang* samples are shown in Table 1. All *doenjang* samples displayed a slightly acidic pH, ranging from 4.97 ± 0.01 to 5.60 ± 0.11. The pH was the highest in control (5.60 ± 0.11) and the lowest in BAR-1 (4.97 ± 0.01). In fermented foods, a pH below 4 to 5 is usually considered to be safe, as most microbial pathogens are unable to survive in such a low pH^17^. The results were consistent with our previous findings in which *doenjang* prepared using different *meju* starter cultures displayed an acidic pH^10^.

### Colour characteristics

Colour is an important physical characteristic of food that catches the attention of the consumer and promotes acceptance. Presently, all six *doenjang* samples showed the yellow surface colour. The maximum yellow surface colour was observed for BAR-1 (24.70 ± 1.40) and minimum for BAR-3 (10.74 ± 0.44). The inner region of all *doenjang* samples displayed a highly intense yellow colour, compared to the outer surface (Table 2). The most likely reason for the more intense inner colour is the Maillard reaction that occur during fermentation and involves several amino acids, sugars, and other components. During fermentation, the reducing sugar enhanced by the action of microbes on the complex carbohydrate is responsible for browning due to the Maillard reaction^18^. The enhanced inner yellow colour value (b* value) of 25.64%, 88.64%, and 80.07% was observed in BAR-1, BAR-2, and BAR-3, respectively, compared to the surface colour value. A similar trend was observed in *doenjang* samples BM15R-1, BM15R-2, and BM15R-3, where the inner colour value was enhanced by 25.55%, 36.20%, and 27.85%, respectively, compared to that of the surface. Both the major preparations, BAR and BM15R, displayed inner colour changes as compared to the surface, however, the most prominent changes were observed in *doenjang* prepared by BAR *meju*. These findings suggested an important role of *A. oryzae* complex in combination with *B. subtilis* and *R. nigricans* in yellow colour development. These results agreed well with the published report, indicating the different colours of *doenjang* samples, based on the starter culture used^12^.

**Table 2:** Colour values of *doenjang* samples fermented with various *meju*.

Side	*Doenjang* samples	Hunter’s value		
		L*	a*	b*
Surface	BAR-1	38.19 ± 0.66	7.62 ± 0.08	13.49 ± 0.89
	BAR -2	38.18 ± 1.59	5.48 ± 0.74	11.36 ± 1.42
	BAR -3	35.65 ± 1.60	6.01 ± 0.23	10.74 ± 0.44
	BM15R-1	51.16 ± 0.43	6.73 ± 0.52	22.23 ± 0.78
	BM15R-2	51.95 ± 0.95	6.47 ± 0.58	21.74 ± 2.80
	BM15R-3	51.54 ± 0.72	7.60 ± 0.48	24.70 ± 1.40
Inside	BAR-1	44.35 ± 0.63	8.72 ± 0.27	16.95 ± 0.60
	BAR -2	45.62 ± 0.54	8.94 ± 0.54	21.43 ± 0.75
	BAR -3	47.84 ± 0.71	8.51 ± 0.55	19.34 ± 1.89
	BM15R-1	54.74 ± 0.17	8.44 ± 0.10	27.91 ± 0.88
	BM15R-2	55.05 ± 0.36	7.99 ± 0.25	29.61 ± 0.99
	BM15R-3	54.34 ± 1.57	8.87 ± 0.55	31.58 ± 1.09

L*, lightness 0–100 (black: 1, white: 100,); a*, redness (−: green, + : red); b*, yellowness (−: blue, + : yellow). Standard L*: 96.43; Standard a*: +0.03; Standard b*: +1.79.

### Free amino acid contents

The contents of individual and total free amino acids of *doenjang* samples are listed in Table 3. Free amino acids present in food products are responsible for the special flavours and are categorised on the basis of their taste characteristics such as sweet, savoury, sour, and bitter^19^. Apart from imparting the flavour to food, amino acids are vital nutrients that have a role as growth factors and stabilizer of nucleic acid^20^. In this study, traditional Korean *doenjang* (control) displayed the highest content of free amino acids responsible for sweet taste (390.20 mg/100 g), followed by BAR-2 (322.50 mg/100 g), and BM15R-3 (320.07 mg/100 g). Of the *doenjang* samples tested for free amino acids related to savoury taste, BAR-2 showed a higher score (285.65 mg/100 g) than traditional *doenjang* (263.20 mg/100 g), followed by BAR-3 (234.49 mg/100 g) and BAR-1 (228.64 mg/100 g). For free amino acids related to bitter taste, BAR-2 (221.73 mg/100 g) showed the highest contents among all tested samples of *doenjang*, followed by BM15R-3 (220.46 mg/100 g) and traditional *doenjang* (209.50 mg/100 g). BM15R-1 displayed the lowest amino acid contents related to bitter taste (125.71 mg/100 g). The highest amount of amino acids related to other tastes was in BAR-2 (226.42 mg/100 g), followed by BAR-1 (196.68 mg/100 g), with lowest content in BM15R-1 (113.63 mg/100 g). The results agreed well with the previous findings of the varied amount of free amino acids in *doenjang* samples and other fermented products^21^. The amount of free amino acids is strongly dependent on the raw material and the microorganisms that participate in fermentation. The variation in the content of free amino acids in different *doenjang* samples can be explained by the fact that they use a different *meju* as a starter culture, and thus the involvement of diverse microorganisms results in consequently varied free amino acids. The results are consistent with our previous report that demonstrated diversification in the free amino acids in different *doenjang* samples with the difference of *meju* as a starter culture^12^.

**Table 3:** Compositions of free amino acids in *doenjang* samples fermented with various *meju.*

Amino acids (mg/100 g)		*Doenjang* samples						
		Control	BAR-1	BAR-2	BAR-3	BM15R-1	BM15R-2	BM15R-3
Sweet taste	Thr	86.80 ± 2.34	41.37 ± 1.80	48.76 ± 1.38	45.14 ± 3.18	29.46 ± 0.45	28.37 ± 0.79	45.11 ± 1.13
	Ser	85.80 ± 3.57	45.51 ± 1.85	55.80 ± 1.34	45.50 ± 3.60	37.49 ± 0.59	44.11 ± 1.23	48.27 ± 1.90
	Gly	53.60 ± 1.52	25.34 ± 0.80	33.09 ± 0.93	29.09 ± 1.85	23.99 ± 0.49	33.21 ± 3.00	28.42 ± 1.77
	Ala	85.00 ± 4.84	82.08 ± 5.52	102.09 ± 4.67	80.24 ± 7.50	77.19 ± 1.36	127.49 ± 7.80	125.21 ± 2.4
	Lys	79.00 ± 2.01	63.66 ± 5.06	82.76 ± 3.12	61.38 ± 6.01	49.98 ± 0.56	67.99 ± 4.87	73.06 ± 0.94
Subtotal		390.20	257.96	322.50	261.35	218.11	301.17	320.07
Savoury taste	Asp	35.60 ± 0.12	31.20 ± 1.93	42.28 ± 0.31	43.34 ± 1.31	13.16 ± 0.20	6.66 ± 1.13	5.83 ± 0.88
	Glu	227.60 ± 2.08	139.36 ± 6.22	181.96 ± 4.64	144.24 ± 1.55	122.23 ± 1.89	158.30 ± 11.43	147.69 ± 5.47
	Cys	0.00	58.08 ± 0.08	61.41 ± 0.02	46.91 ± 0.04	32.66 ± 0.00	43.81 ± 0.09	48.86 ± 0.04
Subtotal		263.20	228.64	285.65	234.49	168.05	208.77	202.38
Bitter taste	Met	13.90 ± 0.21	17.78 ± 1.08	21.70 ± 0.60	19.50 ± 1.16	12.28 ± 0.36	17.59 ± 0.98	22.63 ± 0.64
	Ile	75.60 ± 2.18	65.95 ± 3.42	74.88 ± 2.27	71.98 ± 4.42	43.99 ± 1.02	62.59 ± 1.71	74.70 ± 1.01
	Leu	120.0 ± 1.87	107.30 ± 5.10	125.15 ± 3.25	113.83 ± 6.28	69.44 ± 2.35	98.54 ± 1.72	123.13 ± 2.53
Subtotal		209.50	191.03	221.73	205.31	125.71	178.72	220.46
Other taste	Tyr	82.50 ± 0.05	47.88 ± 0.84	59.82 ± 0.28	27.27 ± 1.20	14.03 ± 1.86	50.84 ± 1.16	27.87 ± 0.07
	Phe	7.10 ± 0.08	63.33 ± 1.54	70.91 ± 2.03	65.14 ± 4.18	41.19 ± 1.12	60.18 ± 0.44	76.46 ± 0.15
	Val	29.60 ± 0.18	36.67 ± 0.88	40.09 ± 3.29	30.66 ± 5.19	22.76 ± 1.00	28.66 ± 4.23	32.76 ± 1.73
	His	5.90 ± 0.01	12.79 ± 0.75	16.83 ± 0.14	13.99 ± 1.58	12.42 ± 0.29	15.93 ± 2.07	19.52 ± 1.61
	Pro	56.0 ± 0.24	36.01 ± 1.45	38.77 ± 1.31	45.25 ± 2.49	23.23 ± 0.58	34.90 ± 2.20	38.08 ± 1.04
Subtotal		181.10	196.68	226.42	182.31	113.63	190.51	194.69

Abbreviations: Threonine; Thr, Serine; Ser, Glycine; Gly, Alanine; Ala, Lysine; Lys, Asparatic acid; Asp, Glutamic acid; Glu, Cysteine; Cys, Methionine; Met, Isoleucine; Ileu, Leucine; Leu, Tyrosine; Tyr, Phenylalanine; Phe, Valine; Val, Histidine; His, Proline; Pro.

### Free sugar contents

**Figure 1:**
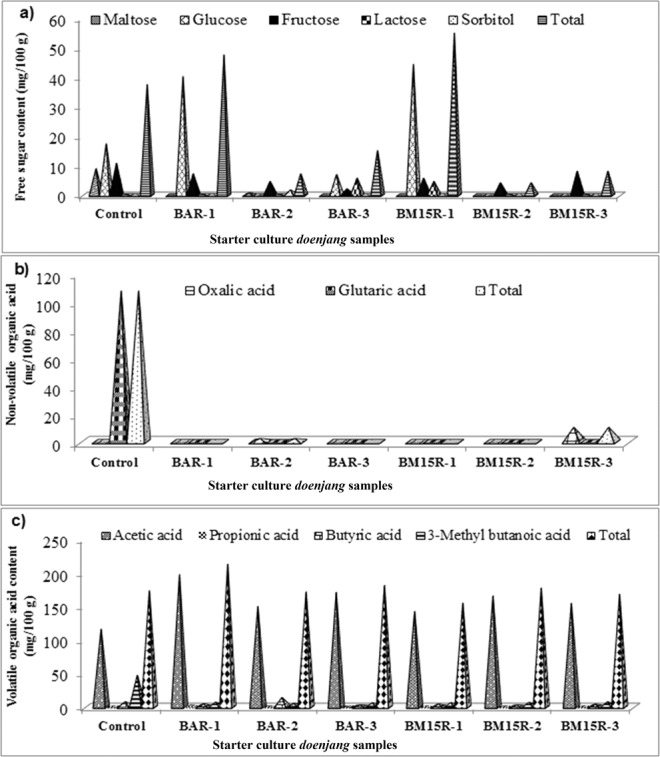
Compositions of free sugars and non-volatile and volatile organic acids in *doenjang* samples fermented with various *meju*.

During fermentation of *doenjang*, various microbial enzymes act on the complex food material and convert it into simple biomolecules such as amino acids, sugar, organic acids, and fatty acids, which are precursors of various volatile compounds^22^. Free sugar content is not only responsible for the nutritive value of food but also important for flavour characteristics. Figure 1a depicts the total free sugar contents of the tested *doenjang* samples. BM15R-1 and BAR-1 displayed the highest total free sugar contents (55.0 mg/100 g and 47.65 mg/100 g, respectively). Among the five tested sugars, fructose was present in all the *doenjang* samples. However, the sugar sorbitol was present only in BAR-2. We speculate that the variations in free sugar contents were due to differences in the *meju* starter culture as a result of the involvement of different microorganisms and the enzyme produced by them^23^. Many reports have demonstrated that the varied amount of free sugars in fermented foods are associated with the involvement of microorganisms. One such a study conducted by our group demonstrated the influence of different *meju* starter culture on the variation in sugar and other metabolites in *doenjang*^12^.

### Non-volatile and volatile organic acid contents

During fermentation, microorganisms convert simple organic biomolecules into various useful products such as organic acids, which impart characteristic tastes, aromas, and functional properties to foods. Organic acids (citric acid and lactic acid) are well known for the improvement of the taste of soy sauce^24^. Knowing the importance of organic acids in fermented foods, we quantified organic acids in the *doenjang* samples, and results are presented in Fig. 1b,c. Among the two non-volatile organic acids tested, oxalic acid was present only in BAR-2 and BM15R-3. On the other side, glutaric acid was not detected in any of the *doenjang* sample. However, glutaric acid was abundant in the control *doenjang* sample.

Among the four volatile organic acids that were tested, acetic acid was present in all the *doenjang* samples with a significantly high amount in BAR-1. In contrast, a high amount of butyric acid was present in BAR-2. Overall, the total volatile organic acids were highest and lowest in BAR-1 and BM15R-1, respectively. These findings indicate the influence of the *meju* starter culture on the production of organic acids, consistent with a previous report concerning the role of starter culture on volatile and non-volatile organic acids in *doenjang*^12^.

### Biogenic amines contents

The most common contamination of soybean-based fermented foods is biogenic amines^25^, which are formed by the decarboxylation of amino acids and serve as a good indicator of food quality^26^. Some biogenic amines, such as putrescine, cadaverine, and agmatine, react with nitrites and are converted into a potential carcinogenic (nitrosamine) and thus pose a serious threat to health^26^. Other than this, aromatic biogenic amines, such as tyramine and 2-phenylethylamine, are the main agents of a dietary-induced migraine and hypertensive crisis^26^. Knowing the importance of biogenic amines in the food, we quantified them in the *doenjang* samples. The results are provided in Table 4. The six *doenjang* samples varied in the amount of biogenic amines. The maximum amount of total biogenic amines was observed in BAR-2 (6.79 mg/100 g), followed by BAR-1 (5.27 mg/100 g). Biogenic amines were lowest in BM15R-3 (0.80 mg/100 g). A negative correlation (y = −3.02 × + 8.40, *r*^2^ = 0.99) between the amount of biogenic amines and the addition of *A. oryzae* complex *meju* was established in *doenjang* samples (Table 4). Among the different biogenic amines, histamine is considered a serious food toxicants and is the cause of histamine poisoning^26^. In all six *doenjang* samples, the histamine level ranged from 0.00 ± 0.00 (BAR-1 and BAR-2) to 0.37 ± 0.02 mg/100 g (BM15R-1). Intermediate values were 0.17 ± 0.03 mg/100 g (BAR-3) and 0.13 ± 0.01 mg/100 g (BM15R-2). The amount of histamine in all the *doenjang* samples was well below the toxicity level^26^, suggesting that the preparations were safe to consume. Biogenic amines in all the samples were also well below the established toxic level^26^. The results are markedly lower than the reported content of biogenic amines in *doenjang*^9,27^. The present results are consistent with our earlier report of a similar profile of biogenic amines in *doenjang* samples prepared by the combination of different *meju*^12^. The present findings also reveal that none of the biogenic amines in the *doenjang* samples were present in high amounts, which might be due to the use of specific starter cultures that have an impact on the inhibition of biogenic amines formation. Results are well supported by the findings of Zaman *et al*.^28^, demonstrating the role of starter culture on the inhibition of biogenic amines.

**Table 4:** Determination of biogenic amines in *doenjang* fermented with various *meju*.

*Doenjang* samples	Biogenic amines (mg/100 g)									
	AGM	TRP	PHE	PUT	CAD	HIS	TYR	SPD	SPM	Total
BAR-1	2.01 ± 0.01	0.05 ± 0.00	0.01 ± 0.00	0.05 ± 0.01	0.00 ± 0.00	0.00 ± 0.00	0.00 ± 0.00	2.17 ± 0.01	0.99 ± 0.04	5.27
BAR-2	1.18 ± 0.01	0.27 ± 0.00	0.72 ± 0.04	0.04 ± 0.00	0.31 ± 0.01	0.00 ± 0.00	0.04 ± 0.01	2.37 ± 0.10	1.86 ± 0.13	6.79
BAR-3	1.60 ± 0.00	0.00 ± 0.00	0.01 ± 0.00	0.16 ± 0.01	0.02 ± 0.01	0.17 ± 0.03	0.43 ± 0.13	0.00 ± 0.00	1.23 ± 0.24	3.62
BM15R-1	0.31 ± 0.00	0.28 ± 0.03	0.01 ± 0.00	0.00 ± 0.00	0.00 ± 0.00	0.37 ± 0.02	0.00 ± 0.33	0.06 ± 0.02	3.92 ± 0.21	4.95
BM15R-2	0.33 ± 0.01	0.46 ± 0.01	0.02 ± 0.00	0.02 ± 0.00	0.02 ± 0.00	0.13 ± 0.01	0.02 ± 0.03	0.04 ± 0.01	0.44 ± 0.10	1.48
BM15R-3	0.26 ± 0.02	0.18 ± 0.02	0.08 ± 0.01	0.02 ± 0.00	0.00 ± 0.00	0.08 ± 0.00	0.06 ± 0.01	0.03 ± 0.00	0.15 ± 0.02	0.80
Mean	0.95	0.21	0.14	0.05	0.06	0.13	0.09	0.78	1.72	

Agmatine; AGM, Tryptamine; TRP, 2-phenylethylamine; PHE, Putrescine; PUT, Cadaverine; CAD, Histamine; HIS, Tyramine; TYR, Spermidine; SPD, Spermine; SPM.

### Cellular antioxidant potential

**Figure 2:**
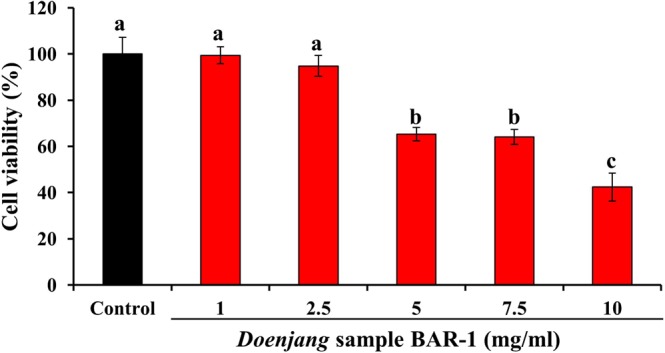
Effect of *doenjang* sample BAR-1 (1–10 mg/ml) on the viability of NIH 3T3 fibroblast cells evaluated by the MTT assay. Results are depicted as mean ± SD of three independent experiments. Values with different letters differ significantly from each other (*P* < 0.05).

**Figure 3:**
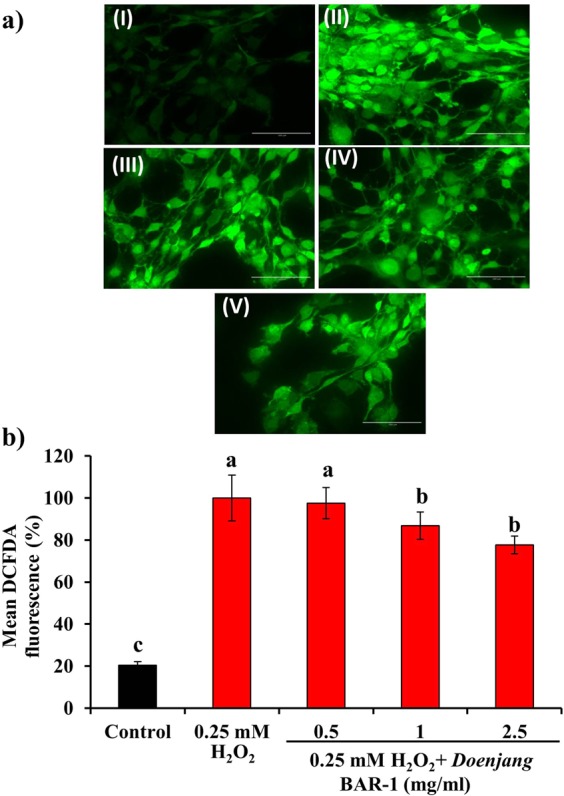
Effect of *doenjang* sample BAR-1 on reactive oxygen species in the NIH 3T3 fibroblast cells challenged by H_2_O_2_. (**a**) DCFDA staining (I) without H_2_O_2_ and BAR-1, (II) with only 0.25 mM H_2_O_2_, (III) with 0.5 mg/ml BAR-1 + 0.25 mM H_2_O_2_, (IV) with 1 mg/ml BAR-1 + 0.25 mM H_2_O_2_, and (V) with 2.5 mg/ml BAR-1 + 0.25 mM H_2_O_2_. All images were taken using an EPI florescence microscope at 40 × magnifications (scale bar = 0.1 mm). (**b**) Fluorescence intensity was determined using Image J software. Each value represents mean ± SD of three independent experiments. Values with different letters differ significantly from each other (*P* < 0.05).

The effect of BAR-1 on the viability of NIH 3T3 cells was evaluated by the MTT assay. The results are depicted in Fig. 2. BAR-1 applied at concentrations up to 2.5 mg/ml had no significant effect on the viability of cells. However, at higher concentrations, the cell viability decreased significantly. The results of cellular antioxidant activity evaluated by DCFDA staining revealed a dose-dependent effect of BAR-1on the inhibition of ROS production in NIH 3T3 cells challenged by H_2_O_2_. A dose-dependent decreased fluorescent intensity of 97.53 ± 7.40%, 86.71 ± 6.40%, and 77.61 ± 4.10% was observed in the cells treated with 0.5 mg/ml, 1.0 mg/ml, and 2.5 mg/ml of BAR-1 respectively, as compared to 100% in only H_2_O_2_ stimulated cells (Fig. 3a,b). Previously, many reports have been described the abundance of phenolics and flavonoids in various soy fermented foods^29–31^. We speculate that the cellular antioxidant activity of BAR-1 is due to the presence of these diverse phytochemicals. Consistent with this, the role of different phenolics and flavonoids as antioxidants by direct scavenging of ROS or by inducing cellular antioxidants has been reported^32^. To the best of our knowledge, no prior study has indicated the cellular antioxidant nature of *doenjang*. However, few reports showing the *in vitro* antioxidant potential of *doenjang*^33^.

### Determination of microbial counts

BAR-1 and BM15R-1 were examined for the viable counts of specific food pathogens. According to Gilbert *et al*.^34^, satisfactory, acceptable, and unsatisfactory limits of detection for coliform bacteria are >2 log CFU/g, 2–3 log CFU/g, and <3 log CFU/g, respectively, in ready-to-eat foods. In both *doenjang* samples, *S. aureus*, *E. coli* O157: H7, *Salmonella* species, and members of the coliform group were not detected (Table 5). The results indicated that the microbial safety of *doenjang* preparations and their good hygienic condition. There are various reasons of the absence of pathogenic microbes. The most prominent is the hygienic manufacturing conditions. Other than this the metabolic profile of prepared *doenjang*, that contains organic acids, which are well known for their antimicrobial properties^35^.

**Table 5:** Determination of foodborne pathogenic bacteria and aflatoxins in various *doenjang* samples.

*Doenjang* samples	Coliforms (CFU/g)	*S. aureus* (CFU/g)		*E. coli* O157:H7 (CFU/g)		*Salmonella* species (CFU/g)		Total aflatoxins (µg/kg)
		BP^2^	API test	MSA^3^	API test	MA^4^	API test	
BAR-1	—^1^	—	—	—	—	—	—	2.17 ± 0.33 (<LOQ^5^)
BM15R-1	—	—	—	—	—	—	—	1.80 ± 0.15 (<LOQ)

^1^Not detected; ^2^Baird-Parker agar; ^3^MacConkey sorbitol agar; ^4^MacConkey agar; ^5^limit of quantification.

### Determination of total aflatoxins

Aflatoxin is a fungal secondary metabolite and the most serious toxicant in food with an adverse health effect even at the lower concentration. According to the guidelines of the Korea Food and Drug Administration, an aflatoxin level of 10 µg/kg (ppb) in food samples is considered hazardous^16^. Food is one of the major sources of aflatoxin poisoning. Hence, we examined the aflatoxin level in the prepared *doenjang*. The level was below the limit of quantification (<5 µg/kg), as described in the Veratox total aflatoxin test kit instructions (Table 5). The absence of pathogenic microbes and the nontoxic level of aflatoxin suggest the prepared *doenjang* to meet the current food safety demand.

## Conclusion

In summary, this study focused on the use of *R. nigricans*-based starter culture, featuring the combined inoculation of bacteria and fungus to improve the quality of *doenjang*. Six different types of *doenjang* samples were manufactured and analysed for their taste and safety characteristics. Furthermore, BAR-1 produced substantial ROS inhibition at the cellular level and was effective in mitigating the harmful effect exerted by ROS. None of the *doenjang* samples contained the biogenic amines above their safety limits. Therefore, it can be concluded that selection of appropriate microorganisms in the proper ratio with *R. nigricans* could be used as a novel starter culture for the production of *doenjang* with enhanced levels of nutritional and functional components and quality characteristics.

## Data Availability

The authors declare that all the data supporting the finding of this study are available within the article and from the corresponding author on reasonable request.
